# Spectrum and Trends of Cancer in Southwestern Uganda from 2012 to 2021

**DOI:** 10.24248/eahrj.v8i1.749

**Published:** 2024-03-28

**Authors:** Yekosani Mitala, Raymond Atwine, Brian Ssenkumba, Abraham Birungi, Barbra Tuhamize, Richard Ezinga, Keneth Male, Kabanda Taseera

**Affiliations:** a Faculty of Medicine, Mbarara University of Science and Technology, Mbarara, Uganda

## Abstract

**Background:**

Cancer has become a global public health challenge and the number one cause of premature death. The incidence is increasing globally and more rapidly in low and middle-income countries despite the gross under-registration and challenges in diagnosis. Data about Uganda is mostly from the Mulago cancer registry which may not entirely represent other parts of the country. This study presents the trends of cancer incidence for Southwestern Uganda in a decade (2012 to 2021).

**Method:**

We did a review of records at the Mbarara University histopathology laboratory and Mbarara Regional Referral Oncology Clinics from January 2012 to December 2021 for cancer diagnoses. Records missing patients' age or sex were excluded.

**Results:**

We registered 4197 incident cancers, 51.8% were among males, and 10.7% were among children (0-18 years). The median age was 52 years and the interquartile range was 35-67 years. The most commonly diagnosed cancers were prostate cancer (13.46%), ocular surface carcinoma (11.03%), cervical cancer (10.55%), head and neck cancers (7.31%), esophageal carcinoma (6.79%), lymphomas (5.36%), retinoblastoma (5.31%), and breast cancer (5.00%) in descending order. Retinoblastoma was the most common cancer among children.

**Conclusion:**

The cancer pattern in Southwestern Uganda has changed and the numbers diagnosed each year are increasing. Prostate cancer is the most common diagnosed cancer overall, and the commonest cancer among men. Among women, cervical cancer is the most common cancer, followed by ocular surface carcinoma, and breast cancer. Retinoblastoma is the most common cancer among children. The trend of Kaposi sarcoma has plummeted together with ocular surface carcinomas. The cancer trends seem to be influenced by the presence of diagnostic and treatment expertise in the region.

## BACKGROUND

Globally, the cancer burden is increasing and this is associated with the changing patterns and trends of different cancers for which the effects are more pronounced in the low- and middle-income (LMICs) countries as evidenced by the increasing incidence and mortality rates respectively.^1,2^ In 2008, approximately 12.7 million new cancer cases and 7.6 million cancer deaths were reported with the majority being observed in the LMICs.^3^ In 2020, global cancer incidence of 19.3 million cases and 10 million related deaths were noted indicating a 34.2% and 24% increment in the incidence cases and deaths, respectively compared to that observed in 2008. Africa alone contributed up to 5.7% and 7% of these increments to incidence and mortality, respectively. These number are expected to further increase and it is predicted that about 28.4 million new cases will be reported by 2040. This predicted trend has been attributed to the changing risk factors and this is a clear indication of the increasing global cancer burden.^1,4^ According to the global cancer observatory, Uganda recorded 34,008 cancer cases and 22,992 cancer-related deaths in 2020 which showed an increment of 4.1% in cancer cases and 5.1% in mortality, respectively compared to the 2018 report.^3^ However, the above data may not truly represent the cancer burden in other regions of Uganda as most of this is information was picked from the Mulago cancer registry which collects its data from the central region only and Gulu cancer registry. This is probably due to the fact that other regions lack organized cancer registries to feed into the national statistics. Owing to the regional differences in major cancer risk factors, the burden of cancer may be different from that reported nationally. Central among the major cancer risk factors is human immunodeficiency virus (HIV), for which Southwestern Uganda has a relatively higher prevalence than the central business district of Kampala.^5^ This is in addition to other sociodemographic risk factor differences and genetics of the catchment areas.

Globally, breast cancer has surpassed lung cancer as the most diagnosed cancer among adults.^3,4^ The lung is the leading cancer site in males, comprising 17% of the total new cancer cases followed by prostate, colorectal, stomach, and liver.^3,6^ In Africa, the most common cancers are infection and poverty related cancers including Kaposi sarcoma (KS), cervical cancer, liver, and stomach cancer although current data reveals a paradigm shift to life style related cancers.^7–10^ This paradigm shift has created a double burden of infections and cancer in most LMICs like Uganda.^9^ In men, the commonest cancers are prostate, liver, colorectum, lung, and Non-Hodgkin's lymphoma (NHL) while in women, the most common are cancers of the breast and cervix, colorectum, liver, and ovary.^1,11,12^ In East Africa, the commonest cancers are; cervical, breast, prostate, colorectal, and esophageal cancer.^12^ In men the commonest are prostate, KS, colorectum, Non-Hodgkin's Lymphoma (NHL) and esophagus while in women the commonest are cervix, breast, colorectum, esophagus and ovary.^12^ The commonest causes of cancer deaths in Africa are cancer of the breast, cervix, liver, prostate and lung in that order, while in East Africa the commonest cancer death is caused by cervix followed by breast, esophagus, prostate and liver.^13^

In Uganda, the commonest cancers are cervical cancer (20.5%), KS (11.3%), breast cancer (7.8%), prostatic adenocarcinoma (7.0%), and NHL (6.9%).^13^ In males, the commonest cancers are KS (17.1%), prostate (16.3%), esophagus (8.9%), liver (8.6%) and NHL (8.5%) in that order while in women, the commonest is cervical cancer (35.7%), followed by breast (13.5%), KS (7.0%), NHL (5.7%) and esophageal cancer (4.2%).^13^ In southwestern Uganda, the only available data is two decades old, and may not be representative of the current situation in the region. It shows that the commonest cancers among men are Kaposi Sarcoma followed by stomach, NHL, prostate and penis while in females the commonest is cervix followed by breast, NHL, stomach and KS.^17^ Away from variations in cancer risk factors, the change in cancer trends and spectrum could be a result of improved cancer screening, diagnostics, and registry in most countries in LMICs.^11,14,15^ Several countries have developed histopathology diagnostic services and radiological imaging which facilitate tumor detection. The Ugandan government currently has a number of histopathology laboratories including at the Central public health laboratory (CPHL), Mulago, Uganda Cancer Institute (UCI), and Mbarara University histopathology laboratory. More ably than before, cancer can be more accurately diagnosed with the aid of techniques like immunohistochemistry, and cytochemistry, among others. The Mbarara University Histopathology laboratory serves the Mbarara regional referral Oncology clinic, a subsidiary of UCI that serves the Southwestern region. This study therefore is availing information on the current burden of cancer in the southwestern Uganda. Results of this study will act as a benchmark for starting a local cancer registry and guide policy makers in regard to resource allocation geared towards cancer prevention and treatment in the region.

## METHODS

### Study Setting

The study was carried out from Mbarara University histopathology laboratory and Mbarara Regional Referral Oncology Clinics. Mbarara university histopathology laboratory and Mbarara regional referral oncology clinic are regional referral centers that serve the southwestern part of Uganda. The centers serve a population of over four million people and the catchment area comprising of the following districts; Mbarara, Bushenyi, Ntungamo, Kiruhura, Ibanda, Buhweju, Rubirizi, Mitooma, and Isingiro. The hospital also receives patients from Kabale, Masaka, Fort Portal, and neighboring countries like Rwanda and Tanzania and the Democratic Republic of Congo.

### Study Design

This was a retrospective cross-sectional study that involved auditing all records at the Mbarara University histopathology laboratory and Mbarara Regional Referral Oncology clinics. We conducted a review of the laboratory register, archived request forms and patient records at the Oncology Clinic from January 2012 to December 2021 for cancer diagnoses.

### Study Population

The study involved patients of all ages with a histological diagnosis of cancer that were registered during the study period.

### Inclusion Criteria

All patients in the laboratory register with a histological diagnosis of cancer.

### Exclusion Criteria

All patients in the laboratory register with a histological diagnosis of cancer whose report could not be retrieved from the archives.

Patients with cancer on treatment at the oncology clinic who were diagnosed from other laboratories other than Mbarara University Histopathology Laboratory.

### Sampling Technique

Convenient non-probability sampling was used. Records were reviewed consecutively as they were identified in the register and retrieved from the archives.

### Data Collection Approach

We reviewed the histology laboratory register to identify all cases with a cancer diagnosis. We then retrieved the laboratory request forms corresponding to the different cases. Biodata, cancer types, grades (whenever found), HIV status (whenever recorded), and cancer site were abstracted on a data capture form developed for this study. Those that lacked patient age, sex and site of tumor, were traced back to the oncology clinics so that the missing information could be obtained from their other hospital records. Despite the effort to try and collect as much information as possible, a lot of patient hospital files were also missing vital information and many could not be traced in the records. Data was double entered in a data entry screen which was created in Epi Info software version 7 (CDC). The data was then transferred into Excel, cleaned, coded, and analyzed using STATA version 17 (StataCorp).

### Data Analysis Plan

Discontinuous variables were expressed as proportions/percentages and presented in pie charts, while the mean was used for continuous variables. Trends of cancers were expressed as tables and line graphs. No associations were determined because of the incompleteness of the data where healthcare workers do not provide information about individual patient's risk factors for the different cancers.

### Ethical Considerations

The study was approved by the Mbarara University of Science and Technology IRB under study number “MUST-2022-681” and also cleared by the Uganda national Council for Science and Technology (UNCST). Site clearance was also sought from the hospital administrators.

## RESULTS

Overall, 4197 cancer diagnoses were observed (Table 2), 51.8% were males, 10.7% of cancers were among children (0-18 years). The Median age was 52 years with interquartile range of 35-67 years. Only 71 charts had HIV status indicated as positive of which 29.6% had KS, 21.1% had Ocular surface carcinoma, and 49.3% had other cancers.

### Age Categorsisation

The frequency of cancer increased with advancing age. Most cancers (29%) were among persons of 65 years and above, followed by those between 50-64 years (25.11%), and lowest in the children 0-18 years at 10.72% (Table 1).

**TABLE 1:** Cancer Distribution Across Different Age Groups

Age groups	Frequency (n)	Percentages (%)
0–18	450	10.72
19–34	556	13.25
34–49	920	21.92
50–64	1,054	25.11
Above 65	1,217	29.00
Total	4,197	100

### Types and Distribution of Cancers Over The Years

**FIGURE 1:**
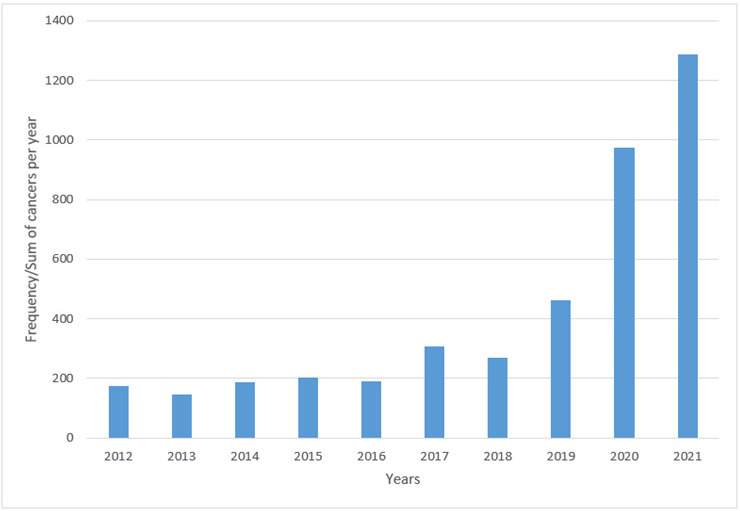
Bar Graph Showing how the Total Number of Cancer Cases Diagnosed Yearly (y-axis) has Varied from 2012 to 2021

Overall, the number of cancers diagnosed each year has been increasing (Figure 1 and Table 3). The most commonly diagnosed cancers over the years are prostate cancer (13.46%), Ocular surface carcinoma (all squamous cell carcinomas (SCC)) (11.03%), cervical cancer (10.55%), head and neck cancers (7.31%), esophageal carcinoma (6.79%), lymphomas (5.36%), retinoblastoma (5.31%), and breast cancer (5.00%) in that order as shown below (Table 2). Among the cervical cancers, 86.23% of the cases were SCC, other subtypes made up the other 13.77% of the cases. Head and neck region included cancers from the oral cavity, tongue, oropharynx, nasopharynx, pharynx, nasal cavity, larynx, and salivary glands. Of the 307 head and neck cancers, 81.11% cases were SCC. Of the 285 esophageal cancers, 72.28% were SCC and 27.72% were adenocarcinomas. Of the 225 lymphomas, 24.44% were Hodgkin lymphomas (HL), 75.56% were Non-Hodgkin Lymphomas (NHL). Of the NHL, 7.65% were Burkitt lymphoma and all occurred in children of which 1 case was ovarian Burkitt lymphoma, and 2 cases were orbital Burkitt lymphoma (Table 2). Except ocular surface cancers, most cancers have had a gradual uptrend since 2012. From 2018, there was a steep uptrend in the numbers of prostate cancer and retinoblastoma, while cervical and esophageal cancers started up trending a year later. While prostate and esophageal cancers maintained an exponential rise through 2020 to 2021, cervical and retinoblastoma had a more moderate rise. Other cancers (gastric, lymphoma, breast, colorectal, and head and neck cancers) have generally maintained a slow upward trend. Ocular surface cancers have to the contrary had a generally downward trend from 2012 to 2014, wavering through 2018 and then gaining a steady slow rise (Figure 4).

**TABLE 2:** Different Cancer Types, Percentages, and Rank in Descending Order

Cancer	Frequency	Percentage	Rank
Prostatic Adenocarcinoma	565	13.46	1
Occular Surface Carcinoma	463	11.03	2
Cervical Cancer	443	10.55	3
Head and Neck Tumour	307	7.31	4
Esophageal Carcinoma	285	6.79	5
Lymphoma	225	5.36	6
Retinoblastoma	223	5.31	7
Breast Cancer	210	5.00	8
Gastric Cancer	197	4.69	9
Colorectal Cancer	157	3.74	10
Sarcoma	142	3.38	11
Skin Tumours	129	3.07	12
Kaposi Sarcoma	120	2.86	13
Melanoma	80	1.91	14
Penile Cancer	78	1.86	15
Hepatocellular Carcinoma	65	1.55	16
Ovarian Carcinoma	57	1.36	17
Vagina and Vulva	51	1.21	18
Endometrial Carcinoma	42	1.00	19
Leukaemia	40	0.95	20
Others	35	0.83	21
Metastatic Carcinoma	35	0.83	22
Urinary Bladder Tumour	32	0.76	23
Intra-Abdominal	27	0.64	24
Thyroid Carcinoma	27	0.64	25
Anal	26	0.62	26
Choriocarcinoma	26	0.62	27
Pancreatic Cancer	21	0.50	28
CNS Tumours	20	0.48	29
Cholangiocarcinoma	13	0.31	31
Gall Bladder Cancer	13	0.31	32
Lung Cancer	11	0.26	33
Small Intestine Adenocarcinoma	11	0.26	34
Wilms Tumour	9	0.21	35
Multiple Myeloma	8	0.19	36
Renal Cell Carcinoma	5	0.12	37
Testicular Tumours	1	0.02	39
**Total**	**4,197**	**100**	

**TABLE 3:** The Distribution of Different Cancer Types Over the Years

Cancer	2012	2013	2014	2015	2016	2017	2018	2019	2020	2021	Total
Anal	0	2	0	2	1	0	0	9	3	8	25
Breast Cancer	3	7	14	24	10	22	17	18	43	52	210
Cervical Cancer	9	9	17	18	19	23	26	19	136	167	443
Cholangiocarcinoma	0	0	0	0	0	1	0	0	8	4	13
Choriocarcinoma	1	1	3	2	1	3	3	4	8	0	26
CNS Tumours	0	0	1	0	0	5	1	7	2	4	20
Colorectal Cancer	4	4	6	7	13	15	10	15	40	43	157
Endometrial Carcinoma	1	2	4	1	5	2	1	5	7	14	42
Oesophageal Carcinoma	1	4	8	2	5	12	5	9	92	147	285
Gall Bladder Cancer	0	1	0	1	0	2	1	1	5	2	13
Gastric Cancer	4	6	10	9	6	16	8	14	55	69	197
Head And Neck Tumour	8	16	23	14	14	28	34	46	61	64	308
Hepatocellular Carcinoma	0	1	1	2	1	1	2	0	23	34	65
Intra-Abdominal	0	1	5	3	2	3	4	5	3	1	27
Kaposi Sarcoma	9	11	17	9	8	16	20	8	11	11	120
Leukaemia	0	0	0	1	1	2	1	25	4	6	40
Lung Cancer	0	0	0	0	1	0	0	0	6	4	11
Lymphoma	7	14	10	8	2	12	23	37	48	64	225
Melanoma	4	6	3	5	1	4	8	16	16	17	80
Metastatic Carcinoma	0	0	3	1	0	2	0	7	8	14	35
Multiple Myeloma	0	0	0	0	1	3	0	3	1	0	8
Occular Surface Carcinoma	103	39	32	49	30	41	34	36	45	54	463
Others	0	0	1	0	1	7	4	4	8	10	35
Ovarian Carcinoma	6	4	3	5	2	4	3	8	11	11	57
Pancreatic Cancer	0	0	2	5	0	0	0	1	5	8	21
Penile Cancer	1	1	1	8	6	2	6	16	17	20	78
Prostatic Adenocarcinoma	1	2	4	6	28	43	34	81	149	217	565
Renal Cell Carcinoma	0	0	1	0	1	0	0	0	0	3	5
Retinoblastoma	0	7	0	0	1	0	0	29	83	103	223
Sarcoma	1	1	6	8	8	9	7	11	37	54	142
Skin Tumours	9	4	7	7	10	16	10	14	16	34	127
Small Intestine Adenocarcinoma	1	1	0	0	1	0	0	2	3	3	11
Testicular Tumours	0	0	0	0	0	1	0	0	0	0	1
Thyroid Carcinoma	0	0	2	4	0	1	4	3	5	8	27
Urinary Bladder Tumour	0	1	0	1	7	4	1	2	5	11	32
Vagina And Vulva	0	2	2	1	4	6	1	8	8	19	51
Wilms Tumour	0	0	0	0	0	0	1	0	0	7	9
**Total**	**173**	**147**	**186**	**203**	**190**	**306**	**269**	**463**	**973**	**1,287**	**4,197**

### Cancer Distribution by Sex and Age

**FIGURE 2:**
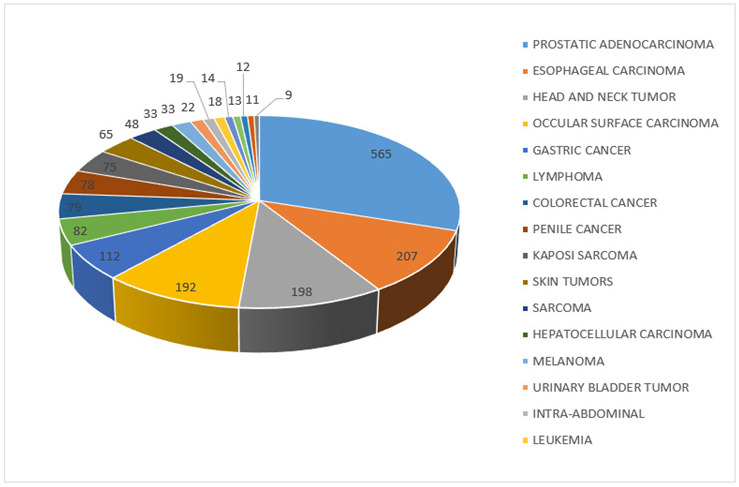
A Pie Chart Showing the 20 Common Cancers in Adult Males

Among adult males, prostate cancer was the commonest cancer. Esophageal cancer was second, followed by head and neck cancer, cancers of ocular surface, gastric cancer, lymphoma, colorectal, and penile cancer (Figure 2). Kaposi sarcoma has significantly reduced and now ranks 8^th^ among men as seen in Table 5 of supplementary documents.

**FIGURE 3:**
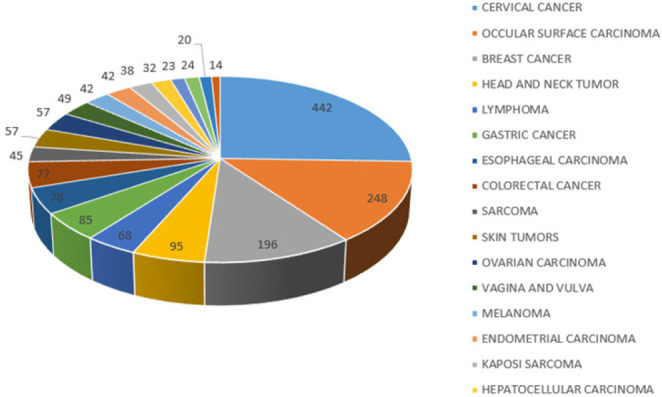
A Pie Chart Showing the 20 most Common Cancers in Women

Among the adult females, cervical cancer was the most common cancer, followed by ocular surface cancer, breast cancer, head and neck, gastric, esophageal and colorectal cancer in that order as displayed in Figure 3. Head and neck cancers were mostly SCC from the oral cavity, tongue, pharynx, larynx, nasal cavity, and other cancers of the salivary glands and jaw. Kaposi sarcoma was even much rarer in women than in men ranking 15^th^ overall among women. Other cancers among women are shown in Table 6 of supplementary documents.

**FIGURE 4:**
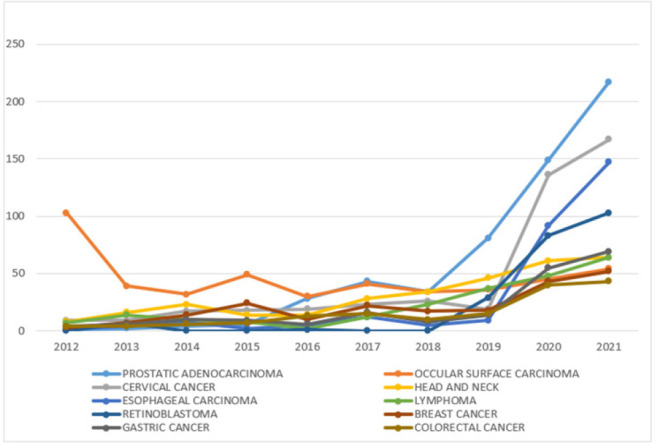
A Line Graph Showing Trends of the Top 10 Common Cancers Overall.

**FIGURE 5:**
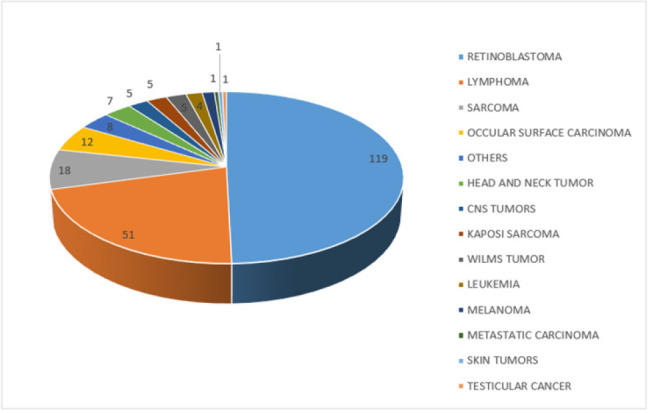
A Pie Chart Showing Childhood Cancers among Boys

**FIGURE 6:**
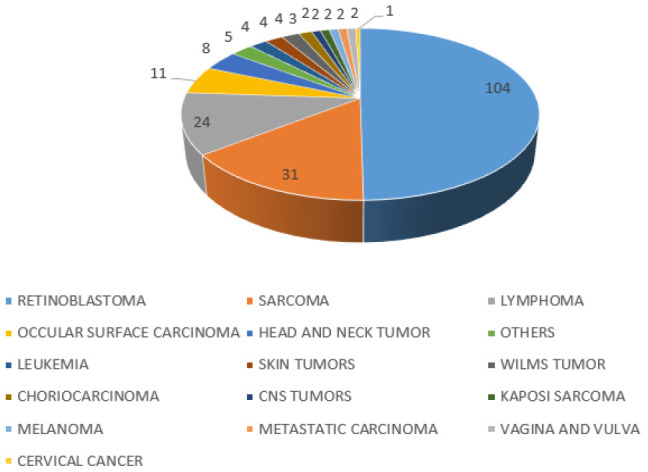
A Pie Chart Showing Commonest Tumors in Girls

Retinoblastoma was the commonest childhood cancer overall and by sex (Table 8, Figure 5-6), followed by lymphomas, sarcoma, ocular surface carcinoma, and head and neck carcinomas (Table 8). Among the lymphomas, 32.00% were HL, 68.00% were NHL of which 17.33% were Burkitt lymphomas (BL). Among the BL, 23.08% were girls and 76.92% were boys. Of the 49 sarcomas, 28.57% were osteosarcomas, 28.57% were rhabdomyosarcomas, and the other sarcomas (liposarcomas, Ewing's sarcoma, chondrosarcomas, and dermatofibrosarcoma protuberans) constituted 42.86%. All Ocular surface cancers (23 cases) were SCC, of which 52.17% were boys, and 47.83% were girls. Among the head and neck cancers, 93.33% were SCC, and 1 (6.67%) was mucoepidermoid carcinoma. The head and neck cancers were equally distributed among boys and girls. One case of cervical cancer was observed in an emancipated minor of 18 years who had a parity of 2 (Table 4 and 8). Among persons living with HIV (PLHIV), KS was the commonest cancer, followed by ocular surface cancer, penile carcinoma, cervical and lymphoma (Table 9 in supplementary materials). However, this may be under-reported due to the incompleteness of the data sources.

**TABLE 4:** Childhood Cancers and Their Frequencies Ranked from Highest to Lowest

Cancer Type	Frequency
Retinoblastoma	223
Lymphoma	75
Sarcoma	49
Occular Surface Carcinoma	23
Head and Neck Tumour	15
Others	13
Wilms Tumour	9
Leukaemia	8
CNS Tumours	7
Kaposi Sarcoma	7
Melanoma	5
Skin Tumours	5
Choriocarcinoma	3
Metastatic Carcinoma	3
Vagina and Vulva	2
Cervical Cancer	1
Testicular Tumours	1
**Total**	**449**

**TABLE 5:** Cancers among Men Showing their Frequencies and Ranked in Descending Order

Cancer	Frequency	Rank
Prostate Cancer	565	1
Oesophageal Carcinoma	207	2
Head and Neck Tumour	198	3
Ocular Surface Cancer	192	4
Gastric Cancer	112	5
Lymphoma	82	6
Colorectal Cancer	79	7
Penile Cancer	78	8
Kaposi Sarcoma	75	9
Skin Tumours	65	10
Sarcoma	48	11
Hepatocellular Carcinoma	33	12
Melanoma	33	12
Urinary Bladder Tumour	22	13
Intra-Abdominal	19	14
Leukaemia	18	15
Breast Cancer	14	16
Anal	13	17
Metastatic Carcinoma	12	18
Others	11	19
Lung Cancer	9	20
Pancreatic Cancer	9	21
CNS Tumours	8	22
Multiple Myeloma	7	23
Small Intestine Adenocarcinoma	7	24
Gall Bladder Cancer	5	25
Cholangiocarcinoma	4	26
Renal Cell Carcinoma	4	27
Thyroid Carcinoma	3	28
**Total**		**1,932**

**TABLE 6:** Cancers among Women Showing their Frequencies and Ranked in Descending Order

Cancer	Frequency	Rank
Cervical Cancer	442	1
Ocular Surface Cancer	248	2
Breast Cancer	196	3
Head and Neck Tumour	95	4
Gastric Cancer	85	5
Oesophageal Cancer	78	6
Colorectal Cancer	77	7
Lymphoma	68	8
Ovarian Carcinoma	57	9
Skin Tumours	57	10
Vagina and Vulva	49	11
Sarcoma	45	12
Endometrial Carcinoma	42	13
Melanoma	42	14
Kaposi Sarcoma	38	15
Hepatocellular Carcinoma	32	16
Thyroid Carcinoma	24	17
Choriocarcinoma	23	18
Metastatic Carcinoma	20	19
Leukaemia	14	20
Anal	12	21
Pancreatic Cancer	12	22
Others	11	23
Urinary Bladder Tumour	10	24
Cholangiocarcinoma	9	25
Gall Bladder Cancer	8	26
Intra-Abdominal	8	27
CNS Tumours	5	28
Small Intestine Adenocarcinoma	4	29
Lung Cancer	2	30
Multiple Myeloma	1	31
Renal Cell Carcinoma	1	32
**Total**		**1,815**

**TABLE 7:** Childhood Cancers among Boys (0-18 years)

Cancer	Frequency
Retinoblastoma	119
Lymphoma	51
Sarcoma	18
Occular Surface Carcinoma	12
Others	8
Head and Neck Tumour	7
CNS Tumours	5
Kaposi Sarcoma	5
Wilms Tumour	5
Leukaemia	4
Melanoma	3
Metastatic Carcinoma	1
Skin Tumours	1
Testicular Cancer	1

**TABLE 8:** Childhood Cancer among Girls (0-18 years)

Cancer	Frequency
Retinoblastoma	104
Sarcoma	31
Lymphoma	24
Occular Surface Carcinoma	11
Head And Neck Tumour	8
Others	5
Leukaemia	4
Skin Tumours	4
Wilms Tumour	4
Choriocarcinoma	3
CNS Tumours	2
Kaposi Sarcoma	2
Melanoma	2
Metastatic Carcinoma	2
Vagina and Vulva	2
Cervical Cancer	1

**TABLE 9:** Cancers among Persons Living with HIV

Cancer	Frequency
Kaposi Sarcoma	21
Ocular Surface Cancer	15
Penile Cancer	6
Cervical Cancer	4
Lymphoma	4
Head and Neck Cancer	3
Leukaemia	3
Breast Cancer	2
Colorectal Cancer	2
Melanoma	2
Others	2
Prostatic Adenocarcinoma	2
Skin Tumours	2
Anal Cancer	1
Gastric Cancer	1
Metastatic Carcinoma	1
**Total**	**71**

## DISCUSSION

In our study, prostate cancer was the overall most commonly diagnosed cancer, followed by ocular surface carcinoma, cervical, head and neck, esophageal, lymphoma, retinoblastoma, and breast cancer. This is a similar finding in many SSA countries^16^ and a major deviation from the global cancer statistics that reveal female breast cancer, and lung cancer as the commonest cancers.^8^ This could be as a result of under-reporting of these cancers in our setting and diagnostic challenges when it comes to certain cancers like lung cancers which require special equipment to obtain the specimens. It was observed that most cancers started showing an exponential increase between 2018 and 2019. This is just a year or two years after the Mbarara Regional Oncology clinics were opened. The center is affiliated to Uganda Cancer Institute (UCI) and it provides care for cancer patients in the region. Before it was started, cancer patients used to be referred to UCI in Kampala which is more than 300 kilometers away. With the opening of the Oncology clinics, the different specialists were also attracted to the region. It therefore implies that the trend is influenced by the presence of different medical specialists and cancer treatment services in the region. At the same time, there was the introduction of an online platform used for signing out cases at the Mbarara University histopathology laboratory. Prior to introduction of this platform, the turnaround time (TAT) used to be so long almost 30 days. This platform reduced the turnaround time from months to just a few days.^17^ As a result of the improved TAT, clinicians that used to send samples to private histopathology laboratories started sending them to the Mbarara University histopathology laboratory thus causing the exponential increase to cancer cases diagnosed each year.

Among males, prostate cancer was by far the most frequent cancer, followed by esophageal, head and neck, ocular surface cancer, and gastric cancer. Similarly, prostate cancer was found to be the most commonly diagnosed cancer in a study conducted in Kyadondo County by Bukirwa et., al (2020).^18^ This is attributed to the increased campaign and awareness of prostate cancer signs and symptoms, the adoption of prostate-specific antigen testing together with prostate biopsies in investigating males with obstructive urinary symptoms. This is in addition to the availability of treatment at the cancer center, and the reduced turnaround time. All these could have contributed to the ever-rising number of prostate cases that are being detected. Compared to a similar study conducted 20 years ago in the same region, the trend has significantly changed. In 2002, KS was the commonest cancer among males in the region followed by gastric carcinoma, NHL, prostate, and penile carcinoma.^19^ Most of these were infection-related cancers; human herpes virus (HHV) 8 for KS, *Helicobacter pylori* for gastric, HIV and Epstein Barr virus (EBV) for some NHL, and HPV for penile cancer. Currently, KS ranks 9^th^, lymphoma ranks 6^th^ and penile 8^th^. It is only prostate and gastric that are still among the top 5 ranking cancers with prostate cancer topping the list and gastric being 5^th^. The increase in prostate cancer has been the steepest, starting gradually in 2015, and gaining an exponential rise from 2018 onwards. The main risk factors for prostate cancer are mainly old age (over 50 years) and race with Africans/blacks more likely to suffer from prostate cancer than other racial groupings.^20^ Esophageal cancer and head and neck cancers in the second and third positions, respectively are mostly due to smoking and excessive alcohol consumption. This demonstrates a shift from infection related to lifestyle-related cancers as predicted by a number of authors.^9,21^ Ocular surface carcinoma on the other hand is infection-associated (HPV) and it has clearly been declining as seen in Figure 3. Gastric carcinoma on the other hand is both infection-related and life style related with obesity and diet being major contributors. A similar trend of decline in infection-related cancer and a surge of lifestyle-related cancers has also been observed by the authors of the Cancer Atlas.^16^ Bukirwa et al., (2021) also observed an increase in nasopharyngeal carcinoma (head and neck), esophageal carcinoma, and prostate among others.^18^ The author also noted a decline in ocular squamous cell carcinoma, KS just like we did in our study. This further emphasizes the shift in cancer risk factors from infections to lifestyle cancers although infection-related cancers are still causing significant morbidities.^16^

Among females, cervical is the commonest diagnosed cancer, followed by ocular surface carcinomas, breast carcinoma, head and neck tumors, gastric, esophageal, and colorectal carcinoma. These finding are similar to those that were observed more than 20 years ago by Wabinga where cervical cancer was the most commonly diagnosed cancer among women in this region.^19^ The findings are contrary to the global picture that shows breast cancer as the most common cancer among females.^8^ The seemingly low cases of breast cancer in the region could be attributed to the absence of breast cancer screening services for example mammography. Therefore, a number of cases could be going undiagnosed or even misdiagnosed as infections.^22^ Strikingly, ocular surface carcinoma has surpassed breast, esophageal and lymphoma to be in the second position. This could be as a result of similar causative agents (oncogenic HPV) to cervical cancer, in addition to environmental factors like Ultraviolet light exposure that is also a great contributor to the development of ocular surface carcinoma. Fortunately, the trends in figure 3 and 4 reflect a net decline in its incidence over the recent years similar to what was observed in Kyadondo County in Kampala.^18^ This could be as a result of the commencement of the eye clinics at Ruharo and at Mbarara Regional Referral Hospital Eye Clinic that now ocular surface precursor lesions are detected early and treated accordingly. Breast cancer ranks 3^rd^ although it may not necessarily reflect a net decline in its incidence rather an effect of other competing cancerous conditions. With increasing awareness and availability of different treatment modalities, the numbers of breast cancers are expected to continue rising. Head and neck cancers rank 4^th^ in females lower than that observed in males. This could be attributed to the fact that women are less likely to involve themselves in risky behavior like cigarette smoking and excessive consumption of alcohol which are major risk factors for these cancers. Unfortunately, the incidence seems to be increasing compared to 20 years ago when it was not among the top 5 cancers in women. Gastric carcinoma comes 5^th^ similar to gastric cancer among males although with slightly more cases than that observed in males. Overall, cases of gastric cancers are on the rise as seen on the graph in Figure 3 and 4 for similar reasons as already explained. Whereas KS was very common 20 years back, currently it is on the decline in both males and females and this could be linked to widespread use of anti-retroviral therapy (ART) and reduced incidence of HIV/AIDS.

Among children, retinoblastoma is the overall most common cancer in the region, followed by lymphoma, sarcomas, and ocular surface carcinoma in that order. This is similar to results obtained in Congo^23^ and Mbarara^24^ where retinoblastoma was the commonest childhood cancer, but contrary to Kyadondo and Kenya where KS and Burkitt lymphoma were the commonest childhood cancers, respectively^23^ The findings are also contrary to observations from Kampala cancer registry where KS, Wilm's tumor and Burkitt lymphoma were the commonest observed cancers.^25^ The overwhelming numbers of retinoblastoma in Southwestern Uganda are due to the presence of a dedicated clinics and specialist doctors for retinoblastoma that receives cases from all around the country and across its borders. Surprisingly, the number of leukemias are abnormally low. This is because the pathology department lacks a hematopathologist to confidently diagnose leukemias with the result that most cases are sent to the Kampala for diagnosis. It therefore implies that the incidence of childhood cancers in this region is largely driven by the presence of diagnostic and treatment services in the region as it was also observed by Stoeter et al.^26^

### Study Limitations

Our study was limited by the incompleteness of data. Clinicians often provide incomplete information about the patients address, and absence of key cancer risk factors like HIV serostatus. This greatly limited useful of our data for the purpose of this study and makes the available data less valuable for future use. For the same reasons, the results may have limited generalizability to the population of Southwestern Uganda since the catchment extends to neighboring countries like Rwanda, Congo and even Sudan.

## CONCLUSION AND RECOMMENDATIONS

The cancer pattern in Southwestern Uganda has greatly changed with an upsurge in the trends of specific cancers since it was last reported 20 years ago. The number of cancer cases diagnosed each year is increasing. Overall, prostate cancer is the most commonly diagnosed cancer in Southwestern Uganda, followed by ocular surface, cervical, head and neck, and esophageal cancer cancers. Among children, retinoblastoma was the most commonly diagnosed cancer. The trend of Kaposi sarcoma has plummeted together with ocular surface carcinomas. The number of cancers diagnosed seems to be influenced by the presence of diagnostic and treatment services in the region. With increasing number of cancer cases each year, the demand for cancer services is bound to increase and therefore need for more government investment in cancer diagnostics and treatment services. From the results above, it is clear that we are shifting towards lifestyle-related cancer. This demands for more preventive measures towards these cancers as well as other noncommunicable diseases that are associated with changing lifestyle. Additionally, there is an urgent need to develop a regional cancer registry to aid in tracking all these cancer cases and aid future research.
